# Mapping the Nanoscale Heterogeneous Responses in the Dynamic Acceleration of Deformed Polymer Glasses

**DOI:** 10.1021/acs.nanolett.4c02261

**Published:** 2024-07-17

**Authors:** Hung K. Nguyen, Bede Pittenger, Ken Nakajima

**Affiliations:** † Department of Chemical Science and Engineering, School of Materials and Chemical Technology, Tokyo Institute of Technology, Tokyo 152-8552, Japan; ‡ Bruker Nano Surfaces, AFM Unit, Santa Barbara, California 93117, United States

**Keywords:** plastic deformation, nanoscale heterogeneity, nanorheology AFM, polymer glasses, segmental dynamics

## Abstract

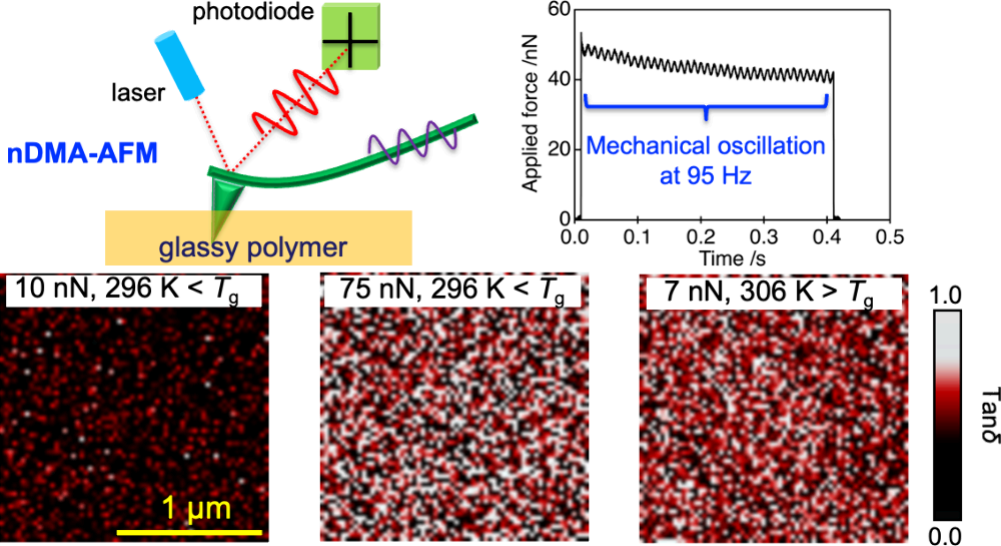

Understanding the evolution of local structure and mobility of disordered glassy materials induced by external stress is critical in modeling their mechanical deformation in the nonlinear regime. Several techniques have shown acceleration of molecular mobility of various amorphous glasses under macroscopic tensile deformation, but it remains a major challenge to visualize such a relationship at the nanoscale. Here, we employ a new approach based on atomic force microscopy in nanorheology mode for quantifying the local dynamic responses of a polymer glass induced by nanoscale compression. By increasing the compression level from linear elastic to plastic deformation, we observe an increase in the mechanical loss tangent (tan δ), evidencing the enhancement of polymer mobility induced by large stress. Notably, tan δ images directly reveal the preferential effect of the large compression on the dynamic acceleration of nanoscale heterogeneities with initially slow mobility, which is clearly different from that induced by increasing temperature.

Disordered glasses are generally formed upon cooling liquids through the glass transition temperature (*T*_g_) without an obvious change in the structural behavior of the molecules.^1,2^ However, such a transition is commonly associated with a substantial slowing of the segmental dynamics of the materials. Despite the extremely slow dynamics, many kinds of amorphous glasses, such as polymer and metallic glasses, can undergo plastic flow rather than brittle fracture under large compressive or tensile stress.^3−5^ It has been recognized that this toughness mechanism is largely related to the fact that many glasses can effectively dissipate large amounts of energy during the plastic deformation, which is supposed to be governed by the relationship between external applied stress and molecular dynamics in the glassy state.^3−11^ Consequently, the influence of mechanical stress on the molecular mobility of amorphous glasses has received great attention in both fundamental and practical studies over the past decades.^5−26^ In theoretical studies, the applied stress is commonly modeled to lower the effective energy barrier that restricts the molecular motions, leading to the increase of molecular mobility of the glass in a similar manner to the effect of increased temperature.^5−9,11^ Thus, the acceleration of molecular mobility in deformed glasses is expected to be a universal phenomenon regardless of how the materials are deformed.^5−8^ This scenario is indeed strongly supported by several simulation results.^13,14,18,19^ For example, Riggleman et al. have proved the similarity in the stress-induced acceleration of the segmental dynamics of a polymer glass under both tension and compression by using double-bridging Monte Carlo and molecular dynamics simulations.^14^

For experiments, several sophisticated approaches based on various techniques, such as nuclear magnetic resonance, optical photobleaching, stress, and dielectric relaxation spectroscopy, have been developed for measuring the molecular mobility of polymer glasses under tensile deformation from the linear to nonlinear regime.^12,15,16,20−24^ Segmental relaxation times of polymer glasses near their *T*_g_ were measured to decrease with increasing stress level, evidencing an acceleration of molecular mobility induced by extension deformation. Such experimental findings are nicely in line with theoretical prediction and simulation results. However, most of reported experiments so far have focused on the macroscopic tensile deformation, in which the enhanced mobility of glasses averaging over a large area can be also attributed to the associated increase of the free volume.^4,14^ It remains challenging to directly measure segmental dynamics of glasses under compression to prove the universal nature of the stress/mobility relationship.

Another important question that remains little known when relying on solely the macroscopic measurements is the effect of local heterogeneities on the stress-induced dynamic enhancement of glasses.^25^ In fact, many studies have shown that the presence of nanoscale structural heterogeneities having packing density fluctuations of short and long characteristic relaxation times is critical to explain various phenomena found in disordered glasses.^1,4,27^ Thus, when subjecting to a large stress, the local dynamic enhancement can be significantly different within heterogeneities, depending on their initial packing behavior.^25,28^ As a result, not only the average dynamic enhancement but also the change of the structural and dynamic heterogeneity and the structure/dynamics relationship induced by the external stress can play important roles in determining the nonlinear properties of glassy materials at the macroscopic scale.^25,29−31^ Recently, a great effort has been made for direct observation of nanoscale structural heterogeneities in multiple glasses by mapping chemical and mechanical responses in linear elastic deformation using different modes of atomic force microscopy (AFM).^32−38^ Unfortunately, most of these AFM modes are not suitable for a quantitative mapping of the dynamic response of materials under plastic deformation.

In this study, we report the direct mapping of the evolution of relaxation dynamics at the nanoscale in a glassy polymer with increasing compressive stress. We employ a new experimental approach based on AFM in nanorheology mode, also known as nanoscale dynamic mechanical analysis (nDMA), which has been recently proved capable of quantifying linear mechanical dynamics of polymers at the nanoscale.^39−41^ The measurements were performed on a solvent casting poly(*n*-butyl methacrylate) (PnBMA) film using probes with a radius of ∼8 nm. Details about the sample preparation and experimental methods are provided in the Supporting Information. The bulk *T*_g_ of PnBMA at 303 K was measured by differential scanning calorimetry (DSC) (Q200, TA Instruments, USA). A DSC curve is shown in Figure S1. Akin to a conventional compression test,^22^ the polymer deformation is expected to change from the elastic to the plastic regime when the stress exerted on the sample by an AFM probe is larger than a critical value. Relying on the loading force–displacement curve, Cappella et al. have proposed that the point where the slope of the curve decreases can be related to a change in the stiffness constant of the sample.^42^ This point was assigned as the transition point. They also demonstrated that at this transition point the glassy polymer is subjected to the transition from elastic to plastic deformation.^42^ Such a correlation is explained in more detail in the Supporting Information. A representative force–displacement curve is shown in Figure 1a, where the point at which the initial slope highlighted by the red dashed line is shifted from the experimental data is supposed to be the transition point, which is observed at a critical force value of ∼30 nN. More evidence of the plastic deformation of the polymer when increasing the applied force to larger than 30 nN is shown later.

**Figure 1:**
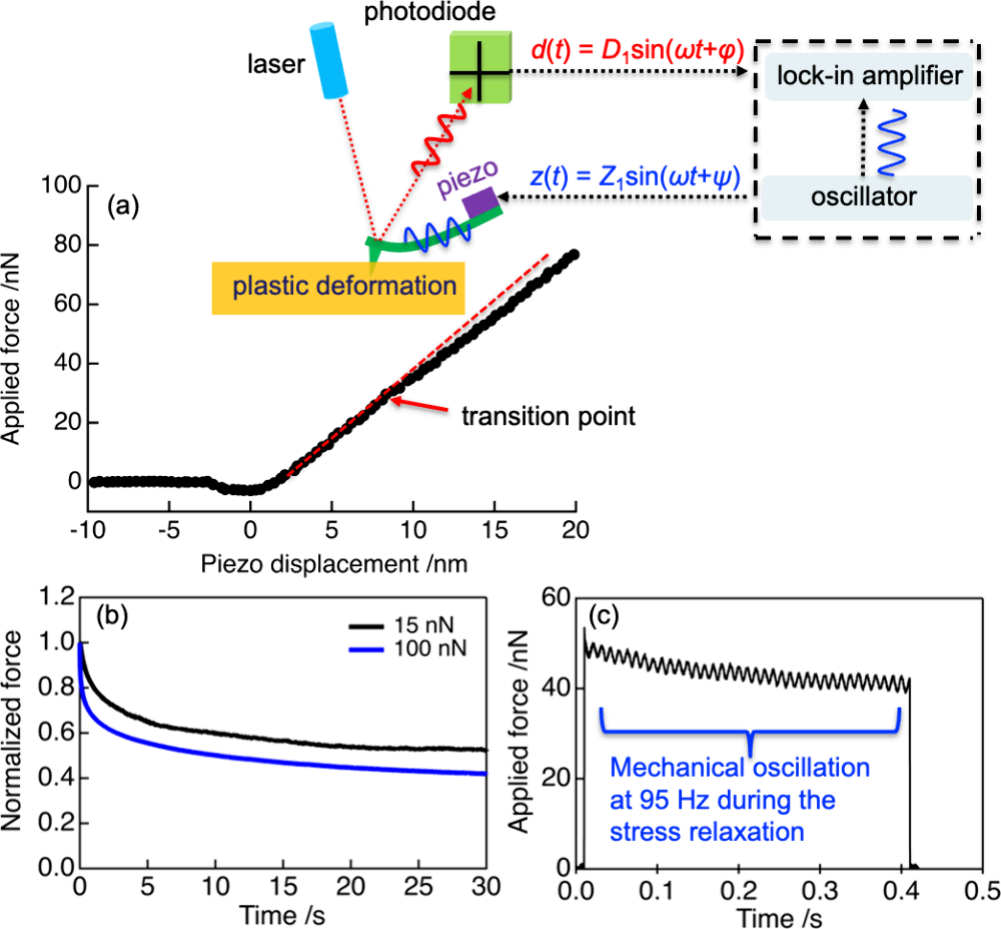
(a) Schematic diagram of AFM for measuring mechanical dynamics of a sample at different levels of applied force. (b) AFM-based stress relaxation of glassy PnBMA at 296 K at different applied forces after a loading time of ∼10 ms. (c) A representative example of an nDMA oscillation curve at 95 Hz during the stress relaxation of glassy PnBMA at 296 K.

Figure 1a also presents a schematic of the AFM approach for measuring mechanical dynamics of the polymer at different levels of the applied force. The probe was approached into the sample surface within a few milliseconds in force-volume mode. At a desired force value, two measuring modes can be conducted. The first mode, denoted as stress-relaxation mode, was performed by detecting the force level as a function of time while the probe and sample positions were kept unchanged. Figure 1b shows representative examples of stress-relaxation curves for the PnBMA film, in which the force data were normalized to the initial maximum forces of 15 and 100 nN. Original curves for the PnBMA film in comparison with data for sapphire and glassy poly(methyl methacrylate) are provided in Figure S2. We can see significant relaxation of the stress over a period of 30 s. Notably, the initial applied force has a clear effect on the resultant relaxation behavior. However, a quantitative characterization of the relaxation dynamics of polymer glasses relying on the stress relaxation curve remains elusive,^16,22,43^ probably due to the presence of a broad distribution of relaxation times of nanoscale heterogeneities in modeling the average mechanical relaxation time.^43,44^

By using nDMA mode, we can bypass the difficulty related to the selecting of a suitable model for fitting the stress relaxation curve.^16,22^ More importantly, this mode enables us to directly visualize the evolution of the dynamic enhancement of the deformed polymer with increasing compression at the nanoscale. In this mode, a driving sinusoidal signal, *z*(*t*) = *Z*_0_ sin(*ωt* + ψ), is applied onto the piezo at the base of the probe when the maximum initial force is reached:^39^ here *Z*_0_, ω, and ψ are the amplitude, angular frequency, and phase of the piezo motion, respectively. The actual oscillation signal of the probe, *d*(*t*) = *D*_0_ sin(*ωt* + φ), was detected at the photodiode, then transferred to the lock-in amplifier for a further analysis: here *D*_0_, ω, and φ are the amplitude, angular frequency, and phase of the probe, respectively. Figure 1c represents a typical oscillation curve of the probe at a frequency of 95 Hz. Other curves measured at 5 Hz are provided in Figure S3. Under mechanical interaction with the sample, the detected oscillation of the probe can exhibit amplitude and phase values different from those of the driven ones. Relying on the amplitude and phase deviations between two signals, we can calculate several dynamic mechanical quantities including dynamic storage (*E*′) and loss (*E*″) moduli and loss tangent (tan δ) of the sample as follows:^39^
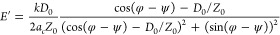
1
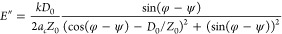
2
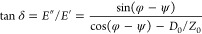
3where *k* is the spring constant of the probe, which is calibrated prior to the nDMA measurement; *a*_c_ is the contact radius between the probe and sample, which is determined by fitting the unloading curve at the end of each pixel point measurement using the Johnson–Kendal–Roberts (JKR) method.^39,41^ The modulation force (*kD*_0_) is maintained to be approximately 2 nN, that is, significantly smaller than the trigger force, to ensure that the probe is in continuous contact with the sample during each pixel point measurement. Figure S4 shows a representative example of nDMA images, including topographic, *E*′, *E*″, and tan δ, simultaneously measured on the PnBMA film with a trigger force of 10 nN and oscillation frequency of 95 Hz. Average values for *E*′, *E*″, and tan δ are 3.3 GPa, 1.1 GPa, and 0.34, respectively, which are quite consistent with mechanical dynamic results for glassy polymers near the *T*_g_.^42^ However, it is important to note that the JKR method is only valid for calculating *a*_c_ when the interaction between the probe and the sample is in the linear elastic regime. Because *E*′ and *E*″, as shown in eqs 1 and 2, depend on *a*_c_, these quantities of the sample cannot be precisely determined in the nonlinear deformation. In contrast, as shown in eq 3, the tan δ quantity depends solely on the oscillation parameters of the probe, providing information about the sample dynamics without knowledge of *a*_c_. In other words, the problem related to the invalidity of the contact model for calculating the contact radius in plastic deformation does not affect the tan δ measurement. Here, we are interested in the segmental dynamics of the sample, which is reasonably quantified through the tan δ measurement in both elastic and plastic deformation.

To confirm the plastic deformation of PnBMA films when subjected to a large force, we directly compared the morphology of the same area before and after nDMA measurements at each applied force. Figure 2 shows these topographic images at different applied forces from 15 to 100 nN: red and blue squares on the top and bottom images, respectively, highlight the sample areas subjected to the nDMA measurement. For low force levels of 15 and 30 nN, the residual plastic deformation was not observed on the highlighted areas after nDMA measurements. In contrast, we can clearly see the arrays of small indents caused by permanent deformation, which appear on the highlighted areas after nDMA measurements at higher force levels of 50 and 100 nN. These results indeed are consistent with the prediction based on the force–distance curve proposed by Cappella et al.^42^

**Figure 2:**
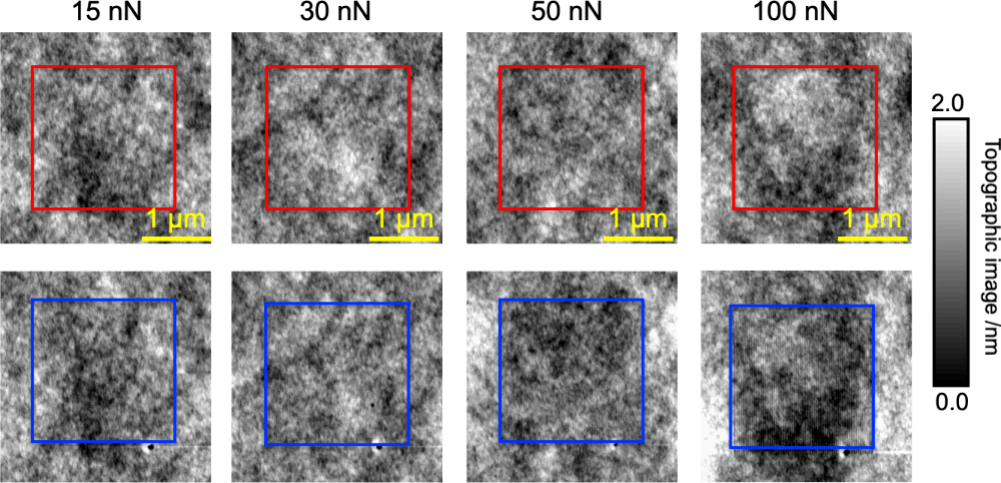
AFM topographic images captured on the areas before (top) and after (bottom) performing nDMA measurements with different applied forces. Red and blue squares highlight the sample areas before and after the nDMA measurement, respectively. Note the array of small indents visible on the bottom row in the area marked by the blue square in the nDMA measurements caused by permanent deformation of the sample (50 nN and 100 nN cases).

Figure 3 shows nDMA tan δ images for the PnBMA film measured at different applied forces from 10 to 100 nN, in which each 64 × 64-pixel image was measured at the oscillation frequency of 95 Hz over an area of 2 × 2 μm^2^. Here, the distance between detected pixel points is selected to be ∼30 nm, relatively larger than the average contact radius between the probe and the sample, which was measured to be ∼6 nm in the linear elastic deformation, as shown in Figure S5. This experimental condition ensures that the deformation of each domain with a size corresponding to the average contact size between the probe and the sample, i.e., of a few to tens of nanometers, is relatively independent from the previously deformed ones. For small forces from 10 to 30 nN, the average value and distribution of the tan δ quantity are nearly independent of the applied force. However, a clear increase of tan δ can be observed by increasing applied force to larger than 30 nN, indicating an enhanced relaxation dynamics of PnBMA under plastic deformation. Although there is no direct correlation between the tan δ quantity measured at a fixed frequency here and the segmental relaxation time of the polymer, our tan δ maps show a relatively dynamic enhancement within different nanoscale domains induced by compressive stress. Therefore, this observation provides direct experimental evidence of the increased mobility of the glassy polymer induced by the compression, in excellent agreement with previous results for samples under tensile deformation,^12,15,16,20,21^ which can also prove the universal nature of the stress-induced mobility enhancement of glassy polymers as predicted in both theoretical and numerical models.^5−8,14,19^

**Figure 3:**
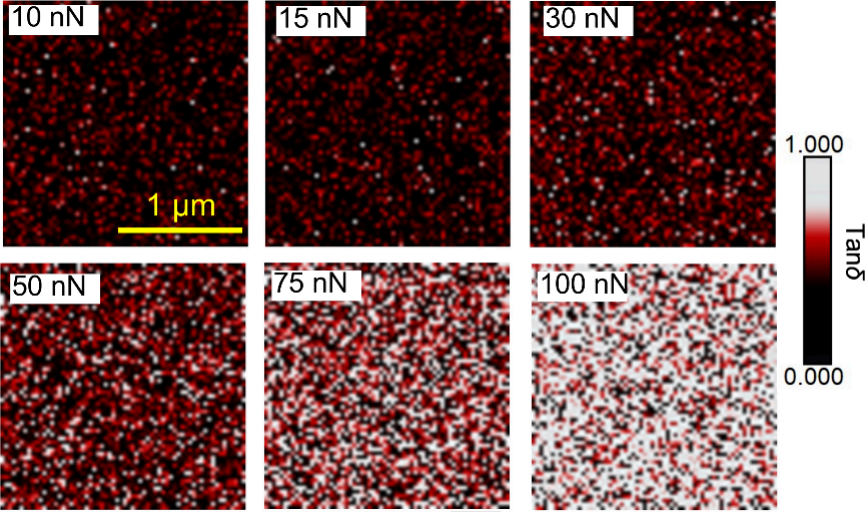
Effect of the applied force on the evolution of mechanical dynamics of glassy PnBMA captured by nDMA tan δ mapping.

More interestingly, tan δ maps shown in Figure 3 at high applied forces evidence the heterogeneous development of dynamic enhancement of nanoscale domains. For example, at the applied force of 50 nN, only a minor portion of domains is observed to exhibit relatively enhanced dynamics compared to those observed at lower forces. Also, at a high applied force of 100 nN, there still remain several domains with slow dynamics (smaller tan δ). In our experimental procedure, the deformation of individual nanoscale domains is not affected by the previous measurements on other domains; that is, the dynamic response of each domain is independent from others. Therefore, it is reasonable to claim that the heterogeneous development observed in tan δ maps with increasing applied stress is correlated to the nanoscale heterogeneous behavior in the structure and dynamics of glassy materials. In fact, because of the difference in the packing behavior of nanostructures, their dynamic responses to applied stress can be substantially different, as recently revealed by Yang et al. using simulation study.^25^

To quantitatively characterize the evolution of the dynamic responses of nanoscale domains at different levels of the applied stress, the tan δ distributions of these domains can be plotted based on the maps in Figure 3. Several representative examples for the tan δ distribution measured at 95 Hz are shown in Figure 4a–e. The reproducibility of the tan δ distribution measured at 95 Hz in both elastic and plastic regimes obtained at different areas is shown in Figure S6, whereas other data measured at 5 Hz are provided in Figure S7. The applied stress is observed to increase both the peak value and distribution of the tan δ quantity of nanoscale domains. In addition, at large stress levels, the tan δ spectrum becomes more asymmetric with broadening at the high-tan δ side, suggesting the presence of at least two contribution components. In fact, the obtained tan δ distributions can be well fitted by using a double-Gaussian function, which probably correspond to two different dynamic modes, as also previously observed in AFM maps of glassy materials.^20^ A comparison of using single- and double-Gaussian functions to fit tan δ distributions is shown in Figure S8 in the Supporting Information. These modes can be supposed to describe the relaxation dynamics of slow and fast nanoscale domains in the glassy polymer.^25^

**Figure 4:**
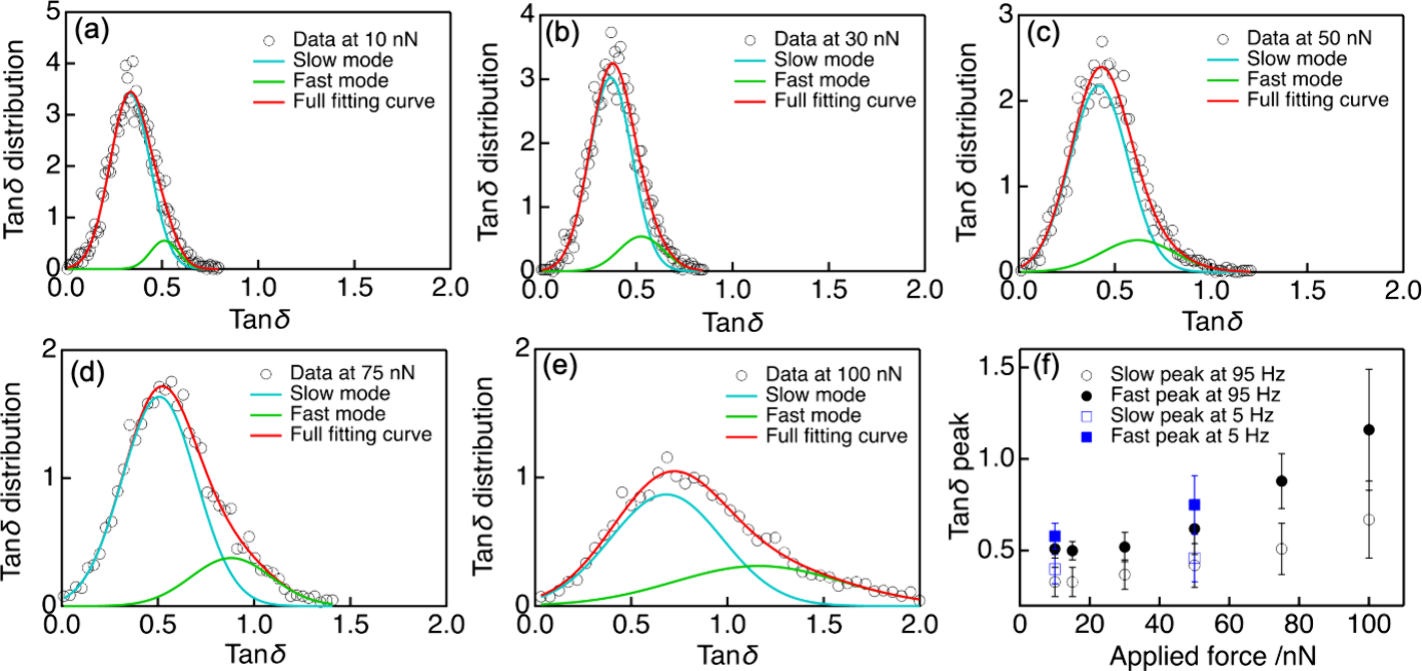
(a–e) Distributions of tan δ maps of a PnBMA film at different applied forces: each red curve represents the fully fitting curve supposedly consisting of two relaxation dynamics modes of slow and fast nanoscale domains in the glassy polymer, which are separately fitted using a double-Gaussian function in cyan and green, respectively. (f) Effect of the applied force on the enhancement of the tan δ peak values of slow and fast modes at different oscillation frequencies.

Figure 4f shows the effect of the applied force on the tan δ peak values of each mode, which are calculated based on the Gaussian fitting curves. The error bars represent the half-maximum width of the fitted Gaussian distributions of each mode. A clear increase of the peak value and the distribution width of tan δ spectra in both modes can be observed when the sample is subjected to the plastic deformation, which is similar for both oscillation frequency values of 5 and 95 Hz. This implies that the stress can accelerate the segmental dynamics of almost nanoscale heterogeneities. However, it is important to note that the increase of the distribution width of tan δ does not indicate that the dynamics of glassy polymer become more heterogeneous induced by the deformation. This is because there is no direct proportional relationship between the relaxation time and tan δ; that is, two domains with a small difference in tan δ when the mobility is slow (such as in small deformation) can exhibit a more significant difference in relaxation time compared to those with a larger difference in tan δ in a more mobile state (such as in large deformation). On the other hand, relying on the Gaussian fitting results, we can quantify the contribution of each mode and calculate their ratios as shown in Figure S9.^45^ Notably, the contribution of the fast mode appears to increase with increasing stress, strongly suggesting that the stress does not evenly accelerate the segmental dynamics of all domains, but preferentially accelerates the mobility of nanoscale domains with initially slow dynamics in accordance with that recently predicted in simulation results.^25^ In other words, the population of fast domains increases with increasing stress levels, which can lead to a reduction in the effective energy barrier for molecular rearrangements in glassy polymers. Therefore, it is expected that the applied stress can ultimately narrow the gap in the mobility of nanoscale domains, making the dynamic responses of the deformed glasses more homogeneous than that of the undeformed systems.^15,16,25^

In theoretical models,^5−8^ the effect of the external stress on the enhanced mobility of glassy materials is generally supposed to be similar to that of the temperature increase. However, experimental studies to evaluate these effects are still limited, whereas recent findings based on the simulation have demonstrated the distinctive roles of the applied stress compared with temperature in accelerating relaxation dynamics of glasses.^10^ To compare the influence of temperature and stress on the segmental dynamics of glassy polymers, we performed nDMA measurements for PnBMA films at several elevated temperatures and different loads. Figure 5 shows a comparison of the effect on the tan δ distributions of elevated temperature (306 K at 7 nN) in the linear elastic regime with lower temperature in the plastic deformation regime (applied force at 75 nN at 296 K). Tan δ distribution at a higher temperature of 313 K together with corresponding tan δ maps are provided in Figure S10. The data measured at high temperatures shown in Figures 5 and S10 evidence an increase of the mean tan δ value with increasing temperature, as expected. In the comparison shown in Figure 5, the temperature is chosen so that the magnitude of the enhancement of segmental dynamics in the polymer (mean tan δ) is similar to that induced by the external stress. However, the distribution of tan δ quantities of nanoscale domains induced by external stress is obviously broader than that induced by the temperature increase. This behavior is also observed for the tan δ distributions measured at higher temperature and applied force, as shown in Figure S10. We also found that (see Figure S9) the temperature has a minor effect on the contribution of slow and fast domains in the tan δ maps. These findings provide direct experimental evidence of the distinctive effects between the applied stress and the temperature on the dynamic responses of nanoscale heterogeneities.

**Figure 5:**
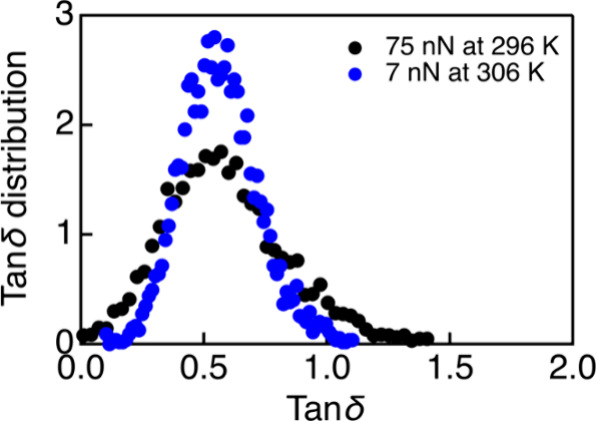
A comparison for the effect of an elevated temperature and external stress on the enhanced dynamics of the PnBMA film.

Using optical photobleaching method,^46^ Ricci et al. have observed a decrease of the characteristic relaxation time of a glassy polymer by more than a decade when increasing the temperature by 10 K, which is comparable to the reduced relaxation time of the same polymer induced by the plastic deformation.^43^ Our results in Figures 5 and S10 are in quantitative agreement with these optical photobleaching data. In addition, our tan δ mapping reveals the heterogeneous nature in the dynamic response of nanoscale domains under the plastic deformation, which probably results from the presence of the intrinsic heterogeneities at the nanoscale in the packing behavior of glassy materials. Such a broad distribution of the tan δ quantity indicates that the reduction of the relaxation times of nanoscale domains can be different from a few factors to a few decades, and depending on the experimental method, each of these ranges can be detected. This might explain the existing discrepancy between different methods in the determinations of the evolution of relaxation times induced by the tensile deformation.^43,44^

In summary, we experimentally investigated the dynamic response of a glassy polymer under nanoscale compressed deformation from linear elastic to plastic deformation. The AFM-based nDMA method is employed to probe the segmental dynamics of the polymer at rheologically relevant frequencies of 5 and 95 Hz. The nDMA tan δ shows the dynamic enhancement of the deformed glassy polymer with increasing compression force, which provides important evidence of the universal nature of the stress/mobility relationship as predicted in both theoretical and numerical models. Importantly, our tan δ mappings enable us to directly visualize the heterogeneous dynamic response of nanoscale domains under plastic deformation, which is also different from that induced by elevated temperature. These results can be therefore important in modeling the nonlinear mechanical deformation of glassy materials with the need of considering the influence of the nanoscale heterogeneities in their packing behavior.

## Supporting Information Available

Sample preparation and experimental methods, nDMA images, and additional experimental data (PDF)
