# Supplementary material for: Mapping the Nanoscale Heterogeneous Responses in the Dynamic Acceleration of Deformed Polymer Glasses
Source: Nano Lett. 2024 Jul 17;24(30):9331–6. doi: 10.1021/acs.nanolett.4c02261 (PMC11299223; doi:10.1021/acs.nanolett.4c02261)
Supplement: Supplementary file 1 — nl4c02261_si_001.pdf [file nl4c02261_si_001.pdf]

Supporting Information

Mapping the Nanoscale Heterogeneous Responses in the Dynamic Acceleration of Deformed Polymer Glasses

Hung K. Nguyen[†*], *Bede Pittenger*[‡], *and Ken Nakajima*[†*]

[†]Department of Chemical Science and Engineering, School of Materials and Chemical Technology, Tokyo Institute of Technology, Tokyo 152-8552, Japan

[‡]Bruker Nano Surfaces, AFM Unit, Santa Barbara, CA 93117, USA

***Corresponding Authors:** nguyen.k.af@m.titech.ac.jp (Hung K. Nguyen) and nakajima.k.aa@m.titech.ac.jp (Ken Nakajima)

1. Materials. Poly(*n*-butyl methacrylate) (PnBMA) with molecular weight M_w = 211 kg/mol was purchased from Sigma-Aldrich. The bulk glass transition temperature (T_g) of 303 K was measured by differential scanning calorimetry (DSC) (Q200, TA Instruments, USA). DSC curves are provided in Fig. S1. For atomic force microscopy (AFM) measurements, PnBMA films with thickness of a few micrometers were prepared by solvent casting of PnBMA/toluene solution into clean silicon substrates. Here, PnBMA/toluene solution with polymer weight fraction of 5 wt.% was used. After the casting, PnBMA films were annealed at 333 K under vacuum for around 3 days to remove the residual solvent from the films. The films were then slowly cooled to 296 K for AFM measurements.

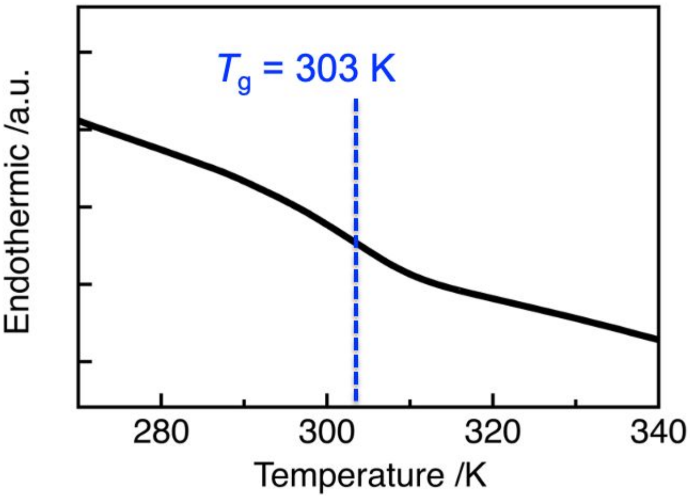

Figure S1. DSC thermogram for PnBMA sample in the second heating scan at a rate of 10 K/min.

2. Determination of transition point. The presence of a point where the slope of force-displacement curve decreases is indeed commonly observed for polymeric materials as assigned in Figure 1a of the main text, although the magnitude of the change strongly depends on the type of materials and temperature.[1,2] In the ideal case, where the deformation of the probe and sample is purely elastic, also without dissipative interaction when the probe is in contact with the sample, there exists a correlation between the deflection of the probe (d_p) and the deformation of the sample (d_s): $d_s = k_p d_p / k_s$, where k_p and k_s are the probe and sample elastic constants, respectively.[1] In the AFM experiment, a relationship between the probe deflection, sample deformation, and sample displacement (Z) can be expressed as: $d_s = Z - d_p$, corresponding to $F = k_p d_p = \{k_p k_s/(k_p + k_s)\}*Z$, where F is the applied force. This relation implies that there exists a linear proportional relationship between the applied force and sample

displacement as long as k_s is a constant. The decrease of the slope can be therefore correlated to a decrease of k_s, *i.e.*, when the sample is subjected to a plastic deformation of the sample. However, in reality, there might exist other factors, such as adhesive and capillary interactions between the probe and sample, influencing the behavior of the slope and transition point. The transition point might not exactly assign the plastic deformation of the sample.

3. Stress-relaxation mode. The stress-relaxation measurements were performed in a Bruker Dimension Icon AFM with Nanoscope 6 controller (Bruker, USA). For AFM measurements, AC200TS probes (Olympus, Tokyo, Japan) were used, of which the nominal spring constant and nominal curvature radius are ~9 N/m and ~7 nm, respectively. The actual values of the spring constant and tip radius were calibrated to be 11 N/m and 8 nm, respectively. In this mode, the force level applied onto the sample was detected as a function of time while the probe and sample positions were kept unchanged. For each force-curve pixel, the ramp size and ramp rate at the loading and unloading sections were 300 nm and 49 Hz, respectively; and the holding time was 30 s. Figure S2 shows several stress-relaxation curves, which were averaged from 20 single pixel curves, measured for PnBMA film at the initial force of 15 and 100 nN in comparison with those for glassy PMMA film and sapphire substrate at the initial force of 100 nN. Here, the sapphire is used as a reference for checking the stability of the probe and sample positions. The stress relaxation curve for the sapphire substrate is almost unchanged after ~20 s, followed up by a monotonic, but slow increase, which can be attributed to the thermal drift of the vertical scanner systems. Nevertheless, such a thermal drift effect can be negligible in discussing our results which were obtained within a few seconds for each probe/sample contact. It is clear that the stress-relaxation was only observed for the case of PnBMA film, for which the measured temperature of 296 K is slightly below the sample T_g, thus the segmental relaxation time is expected to be in the order of measured time duration.[3] In contrast, for the case of PMMA film, the segmental relaxation time is much longer than 30 s, thus the stress-relaxation is not expected to be observed.[4]

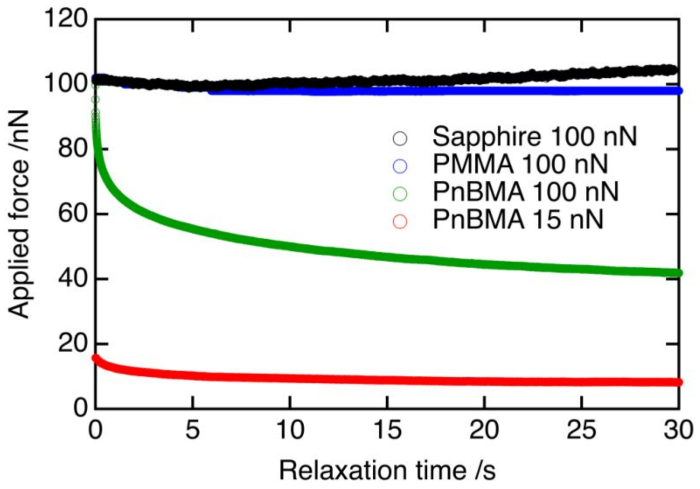

Figure S2. Stress-relaxation curves measured by AFM method for different materials.

4. Nanoscale Dynamic Mechanical Analysis (nDMA) mapping. The nDMA measurements were performed in a Bruker Dimension Icon AFM with Nanoscope 6 controller (Bruker, USA). For each force-curve pixel, the ramp size and ramp rate at the loading and unloading sections were 300 nm and 49 Hz, respectively; the holding times at modulation frequencies of 95 and 5 Hz were 400 ms and 5 s, respectively. Figure S3 shows several oscillation curves of the probe at a frequency of 5 Hz for PMMA and PnBMA films at different applied forces. Figure S4 shows a representative example of 64×64-pixel nDMA images including topographic, E', E'', and tanδ, simultaneously measured on the PnBMA film with a trigger force of 10 nN at the oscillation frequency of 95 Hz over an area of 2×2 μm^2.

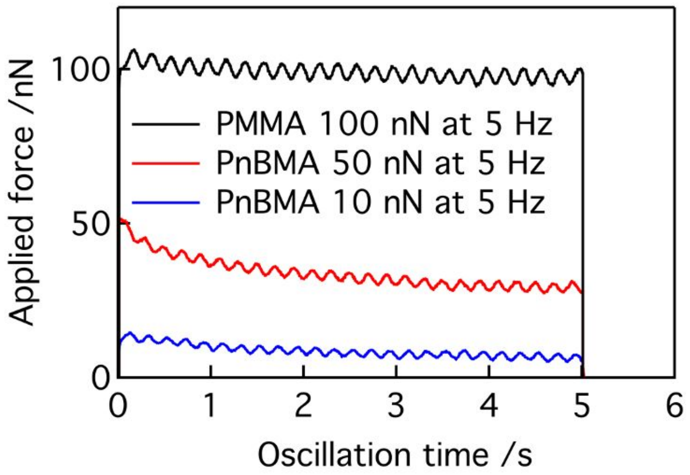

Figure S3. Representative examples of nDMA oscillation curve at 5 Hz during the stress relaxation of glassy PMMA and PnBMA films at 296 K.

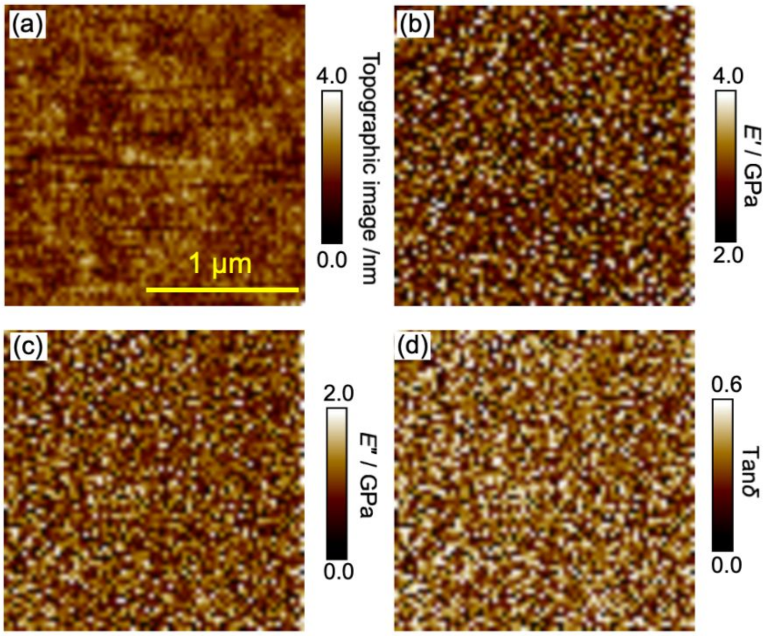

Figure S4. (a) Topographic, (b) storage modulus, (c) loss modulus, and (d) loss tangent images obtained for PnBMA film using nDMA mode in linear elastic regime with a trigger force of 10 nN and oscillation frequency of 95 Hz.

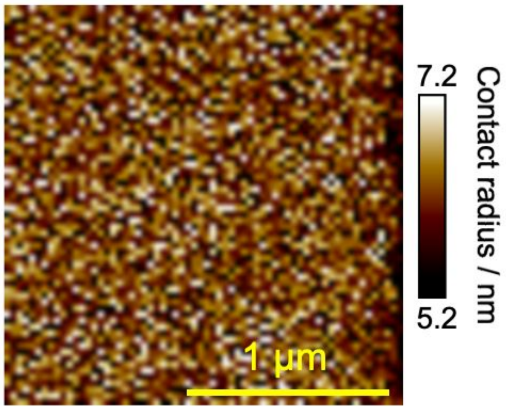

Figure S5. nDMA contact radius map showing the contact radius between the probe and the PnBMA film during the nDMA measurement at a trigger force of 10 nN.

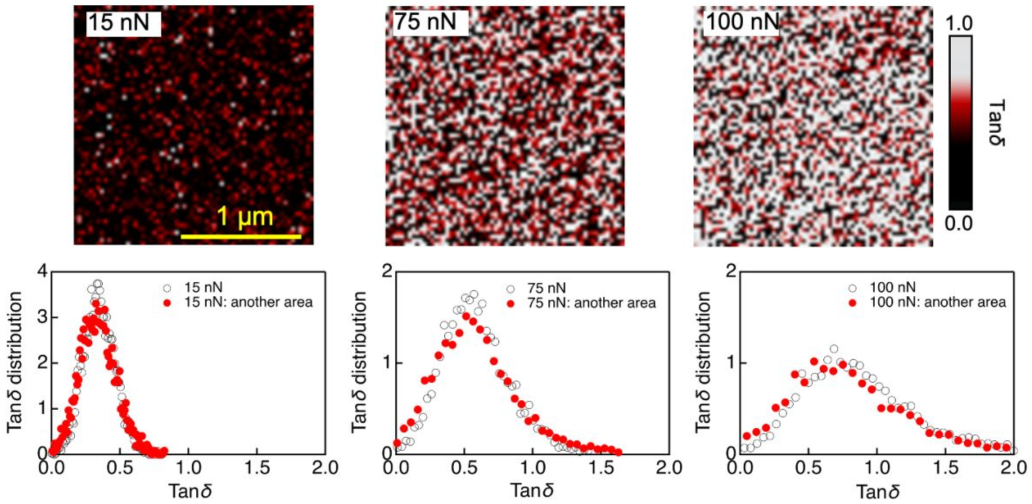

Figure S6. A comparison of tanδ maps of PnBMA film and their distribution measured at different areas under both elastic (15 nN) and plastic deformation (75 and 100 nN). A good reproducibility between two measurements at different areas can be observed.

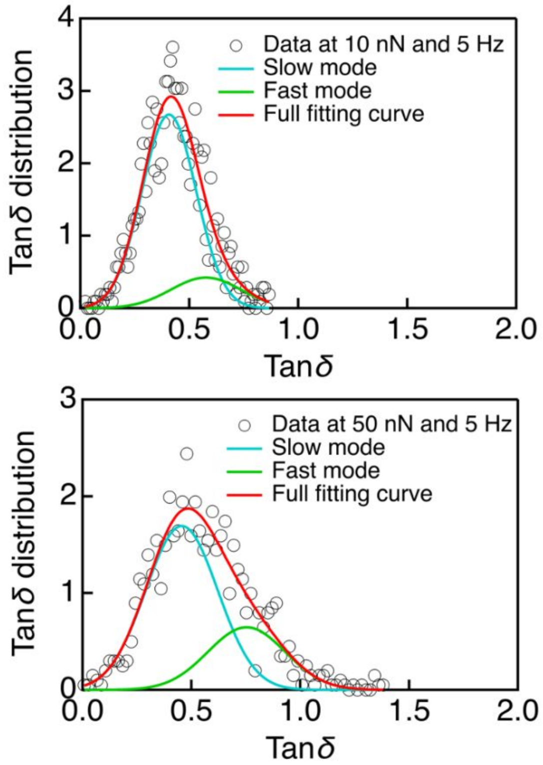

Figure S7. Distributions of tanδ maps of PnBMA film measured at different applied forces measured at an oscillation frequency of 5 Hz: each red curve represents the fully fitting curve supposedly consisting of two relaxation dynamics modes of slow and fast nanoscale domains in the glassy polymer, which are separately fitted using a double-Gaussian function in cyan and green, respectively.

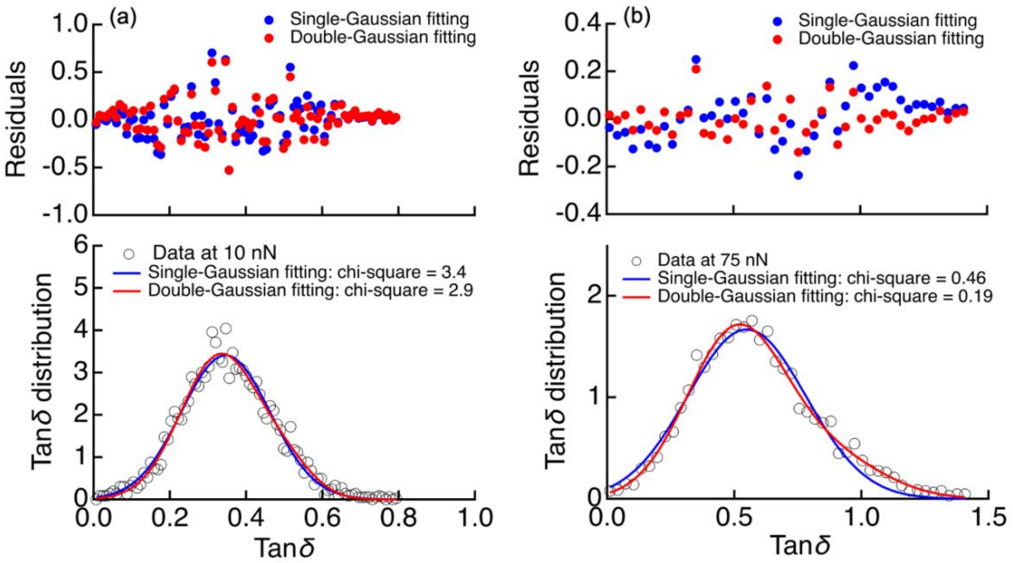

Figure S8. A comparison of using single- and double-Gaussian functions to fit tanδ distributions obtained in (a) elastic (10 nN) and (b) plastic (75 nN) regimes. Both residual and chi-square data suggest that the double-Gaussian function can better describe the dynamic response of nanoscale domains captured by tanδ mapping.

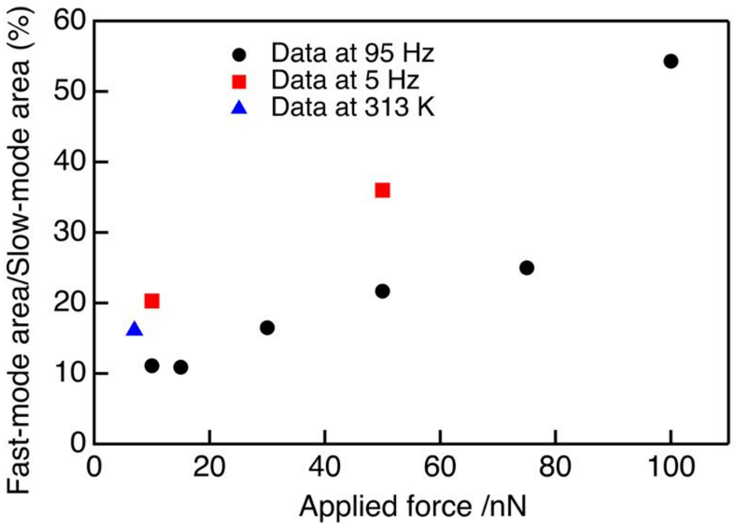

Figure S9. Evolution of the contribution of the fast-mode domains relative to that of the slow-mode domains with increasing stress at 5 and 95 Hz as well as at an elevate temperature of 313 K.

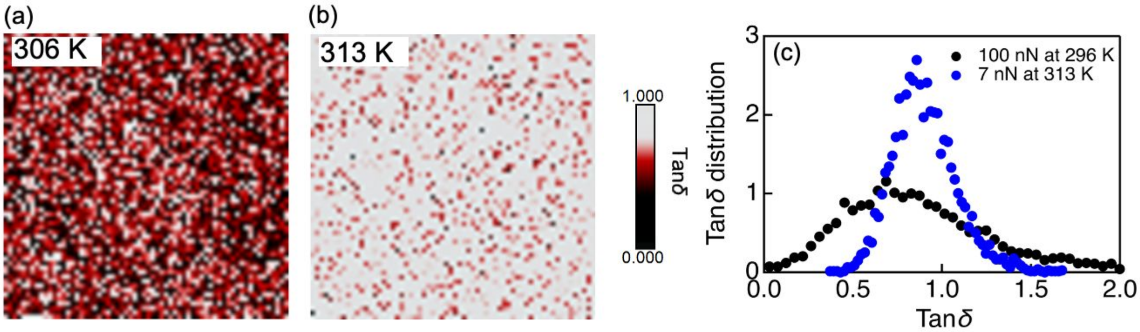

Figure S10. (a,b) Effect of the elevated temperature on the evolution of mechanical dynamics of glassy PnBMA captured by nDMA tanδ mapping at 95 Hz and applied force of 7 nN. (c) A comparison for the effect of elevated temperature and external stress on the enhanced dynamics of the PnBMA film.

(1) Cappella, B.; Dietler, G. Force-Distance Curves by Atomic Force Microscopy. *Surf. Sci. Rep.* **1999**, *34*, 1–104.

(2) Cappella, B.; Kaliappan, S. K.; Sturn, H. Using AFM Force-Distance Curves to Study the Glass-to-Rubber Transition of Amorphous Polymers and their Elastic-Plastic Properties as a Function of Temperature. *Macromolecules* **2005**, *38*, 1874–1881.

(3) Li, X.; Liu, J.; Liu, Z.; Tsige, M.; Wang, S.-Q. Illustrating the Molecular Origin of Mechanical Stress in Ductile Deformation of Polymer Glasses. *Phys. Rev. Lett.* **2018**, *120*, 077801.

(4) Lee, E.-W.; Medvedev, G. A.; Caruthers, J. M. Deformation Induced Evolution of Mobility in PMMA. *J. Polym. Sci. Part B: Polym. Phys.* **2010**, *48*, 2399–2401.
